# Herpes Simplex Virus in the saliva of peripheral Bell's palsy patients

**DOI:** 10.1016/S1808-8694(15)30026-4

**Published:** 2015-10-19

**Authors:** Paulo Roberto Lazarini, Melissa Ferreira Vianna, Mônica Porto Alves Alcantara, Rodolfo Alexander Scalia, Hélio Hehl Caiaffa Filho

**Affiliations:** a Assistant Professor at the Otolaryngology Department of the Santa Casa de São Paulo, Post-Graduation Professor of the Medical School of the Santa Casa de São Paulo; b Otolaryngologist taking the Enhancement Course in Otolarygologist at the Santa Casa de Misericórdia de São Paulo; c PhD – Assistant Professor of Microbiology from the Department of Pathology - the Medical School of the Santa Casa de São Paulo Santa Casa de São Paulo

**Keywords:** Facial palsy, Herpes Simplex, Saliva

## Abstract

The first herpes virus to be described was types 1 and 2, whose denomination is herpes simplex 1 and 2 or HSV -1 and HSV -2. These viruses have specific biological characteristics, such as the ability to cause different kinds of diseases, as well as to establish host's latent or persistent lifetime infections and also of being reactivated, causing lesions that can be located at the same site of the initial primary infection or close to it. It is suggested that this virus reactivation in the geniculate ganglion may be related to Bell's palsy. In this situation, the viruses that would be latent in this ganglion, would suffer reactivation and replication, then be diffused through the facial nerve and its branches, among them the chorda tympani nerve, which by stimulating salivary secretion would enable the identification of the viral DNA in the patients’ saliva. Until recently, a great number of patients was diagnosed as holders of this kind of paralysis, named idiopathic or Bell's palsy. With the introduction of the technique studying the viral DNA by Polymerase Chain Reaction (PCR), several authors have found herpes simplex virus type I DNA in the cerebrospinal fluid, in the lachrymal secretion, in the saliva and in the geniculate ganglia of patients with Bell's palsy.

**Aim:**

observe the occurrence of herpes simplex type I virus using PCR technique in the saliva of patients with Bell's palsy and relating it to the clinical evolution of these cases.

**Methodology:**

We evaluated 38 patients with Bell's palsy submitted to anamnesis, clinical and ENT examination and saliva sampling for viral DNA detection by PCR technique. The control group was ten normal adults.

**Results:**

We found positive viral DNA in 11 cases out of the 38, which corresponded to 29% of the sample. This result was statistically significant if compared to the control group, in which we did not find any positive case.

**Conclusion:**

The end result was that the presence of HSV -1 in the saliva of patients with Bell's palsy indicating that the viral reactivation can be the etiology of this disease. The detection of the virus in these patients’ saliva does not influence the disease prognosis.

## INTRODUCTION

Types 1 and 2 were the first herpes virus described (HHV-1 and HHV-2), called herpes simplex 1 and 2 or HSV-1 and HSV-2. These viruses bear particular biologic characteristics, such as the potential to cause different types of diseases, to establish latent or persistent infections during the host's life time and that of being reactivated causing lesions that may be located on the site of the primary initial infection or near it.

The lesions caused by these viruses were first documented by Hypocrates (460/377 b.C.), who called them herpes, from reptile, because of the skin vesicles. But it was only in 1968, that two types of herpes virus - Simplex virus genus were classified on the basis of their biological and antigenic differences. These observations were fundamental for clinical, serological, immunological and epidemiological studies that peaked at the establishment of the anti-viral therapy, and it also demonstrated the genotypic and phenotypic differences among variants of HHV-1 and 2.

The International Committee on Taxonomy of Viruses (ICTV) classified this virus in the Herpes viridae family, Alphaherpesvirinae subfamily, Simplex virus genera, types 1 and 2. Both HSV-1 and HSV-2 are made up of an electrodense core of 150-153 Kpb double linear DNA strains. HSV-1 and 2 infect different cell types, such as fibroblasts, squamous and mucous cell epithelia, polarized cells of the cylindrical epithelium, glial cells and nerve endings.

These virus show the capacity to establish latent or persistent infection throughout its host's life, changing the latency site according to its subfamily. The major characteristics of the alfa-herpesviridae family members is to establish latency in the sensory nerves. During this latency period, the viral DNA is not completely silent. None of the viral genes expressed during the lytic phase is detected. However, some RNA called latency associated transcripts may be found in high levels. In the same way, these viruses may show the reactivation mechanism, when the virus may be reactivated in peripheral tissues, and this could, at least in part, explain the virus transmission in an asymptomatic period.

Clinically, most HSV-1 is isolated in herpetic lesions of the oropharynx. After the acute or symptomatic phase of virus manifestation, it may remain latent in the sensitive ganglia neurons, as in the facial nerve case, the geniculate ganglion. The viral reactivation in this site may cause an inflammatory process able to trigger a peripheral facial paralysis. This is the main disease that affects the facial nerve and has broad etiology, where systemic, inflammatory and tumoral diseases, among other, may determine it^4^, ^5^.

Clinically the peripheral Facial Paralysis is a disease characterized by the loss of muscle movement in half the face, generating a clear deformity during facial expressions.

Until recently, a large number of patients was diagnosed as carriers of one type of this paralysis, the one called idiopathic or Bell's paralysis. These terms are still used in the literature to characterize those patients with acute signs of peripheral facial paralysis and without an etiological diagnosis defined even after conventional clinical, laboratorial and image investigations^4^.

Starting on the 70's, many studies tried to establish this disease etiology^5^, ^6^, ^7^. Among them, many are the papers that present cases showing some positive serology for herpes simplex virus, varicella-zoster, mononucleosis, mumps, measles, among others^7^. With the creation of the viral DNA study technique with the polymerase chain reaction (PCR), many authors found type I herpes simplex virus DNA in the facial nerve endoneurium fluid, CSF, tears, saliva and the geniculate ganglia of Bell's palsy patients^8^, ^9^, ^10^.

Identifying HSV-1 DNA virus in all these circumstances indicate that the type 1 herpes simplex virus may very well be the major agent responsible for this peripheral facial paralysis (PFP)^11^, ^12^, ^13^, ^14^. In serial studies involving Bell's PFP patients, Furuta et al.^13^ found 17% of HSV-1 PCR positive cases in the saliva for a total of 176 patients investigated. However, Abiko et al., in 16 patients, found 5 (31%) positive tests.

The goal is to detect type 1 herpes simplex virus by PCR in the saliva of Bell's Peripheral Facial Paralysis patients, comparing them to a control group and correlating it to electroneurography and degree of paralysis results.

## MATERIALS AND METHODS

The study was carried out in a tertiary University Hospital and was referred and approved by that institution Medical Ethics Committee.

### Selection of clinical cases

38 consecutive patients were assessed and diagnosed as carriers of Bell's Peripheral Facial Paralysis according to the following inclusion criteria:

a) Acute peripheral facial paralysis with symptoms starting up to seven days after they were first seen at the hospital;
b) Physical and otolaryngological exam without evidences of a determined cause for the PFP.

The control group encompassed 10 normal adults.

## METHOD

a) Medical follow up

The patients were seen as they arrived at the medical facility, were then interviewed and went through general and otolaryngological physical exams.

b) Material harvest

In this visit, saliva was harvested from the patients’ mouth using a disposable sterile syringe without the needle. The saliva was stored in a proper container for the PCR (EPENDORFF).

c) Lab exams

Tests were carried out in order to detect the HSV-1 viral DNA through PCR using specific primers aforementioned for this virus.

d) Statistical analysis

The results obtained both from the clinical and laboratorial views were statistically analyzed.

## RESULTS

38 patients with acute Bell's PFP were assessed, 16 males and 22 females. Of those, 11 (29%) had positive PCR for the HSV-1 virus in the first week of disease development. The positive PCR cases were divided in 5 females (23%) and 6 males (37%).

The age ranged from 6 to 71 years, and 12 of these patients were between 21 and 30 years. Table 1 shows cases distribution according to age and results for the HSV-1 PCR test.

**Tabela 1:** Bell's Palsy case distribution according to age range and the PCR result test for saliva HSV-1 in the first week of the disease - 2004.

Age (years)	# PCR + cases n (%)	# PCR –cases n (%)	# of cases assessed
0-10	0	3 (11)	3
11-20	2 (18)	5 (18)	7
21-30	3 (27)	9 (33)	12
31-40	1 (9)	4 (15)	5
41-50	3 (27)	3 (11)	6
51-60	1 (9)	2 (7)	3
61-70	0	1 (4)	1
71-80	1 (9)	0	1

We could see that in the assessed clinical cases, the stapedial reflex was not present in 21 cases and present in the other 13 cases. Among PCR positive cases, 9 patients (80%) did not have this reflex while only 2 patients (20%) had the reflex present.

Table 2 depicts the facial nerve electroneurography results according to the HSV-1 PCR test.

**Table 2:** Distribution of facial nerve electroneurography results according to PCR for HSV-1 in Bell's Palsy cases.

EnoG (%)	# PCR + cases n (%)	# PCR -cases n (%)	# cases
0-20	2 (18)	4 (18)	6
21-40	3 (27)	6 (26)	9
41-60	4 (36)	2 (8)	6
61-80	0	9 (39)	9
81-100	2(18)	2 (8)	4

Table 3 depicts the degree of facial paralysis according to the House-Brackmann classification (1985) at the patient's admission compared to the HSV-1 PCR test results.

**Table 3:** Distribution of Bell's Peripheral Paralysis Cases according to the degree of paralysis and the PCR test result for HSV-1.

GRADE	# Cases	PCR positive # (%)	PCR negative # (%)
II	2	0	2 (7)
III	10	1 (9)	9 (33)
IV	8	3 (27)	5 (19)
V	11	4 (36)	7 (26)
VI	7	3 (27)	4 (15)

As far as development is concerned, of the 38 patients assessed, 27 were discharged without sequelae and three remained in grade II of the House-Brackmann classification. Two remained in grade V, three patients were decompressed and three did not have clinical follow up.

Of the positive patients, three did not improve and one was referred to surgery, another remained in grade II and another, still, in grade V.

## DISCUSSION

In the acute PFP the medical team has to act quickly in order to establish the most adequate diagnosis and treatment for the patient and thus, avoid future facial motor sequelae. It is of fundamental importance to define the etiology both for treatment purposes and to provide a more accurate prognosis. It is not always easy though, therefore it is important to carry out a careful clinical evaluation and, in many cases, a number of complementary tests.

Bell's palsy in our settings is characterized for being a diagnosis of exclusion. A new situation is arising with modern lab techniques for viral investigation that is making this current concept, which is around since the 90's, broadly modified and the facial nerve involvement in the PFP is better assessed.

In this study, 29% of the Bell's PFP cases had HSV-1 positive test in their saliva. This figure is greater than the one found by Furuta et al., who evaluated 176 patients. Compared to the results from Abiko et al., our data coincide, although his sample was smaller (16 patients). We may believe that these positive cases may have had a virus reactivation process as etiology.

The establishment of this relationship may decisively influence the introduction of antiviral drugs in the treatment of these patients. HSV-1 was not influenced by the age factor in our investigation.

This finding is not broadly discussed in the literature and does not uphold the idea that viral reactivation occurs more frequently in elderly patients. One relevant fact was that we did not find the stapedial reflex in 80% of HSV PCR positive cases. PCR negative cases had equal distribution as far as the presence or absence of this reflex is concerned.

Analyzing this fact, we can postulate that if the virus is reactivated in the geniculate ganglion, migrated through the facial nerve and reached the salivary gland (via chorda tympanis nerve), it is likely that there may have occurred an inflammatory process in the facial nerve tympanic-mastoid segment and, with that, involvement of its stapedial branch (reflex absence) happening more frequently.

As to facial nerve electroneurography, we did not observe significant difference of results in comparison with PCR positiveness for HSV-1 (Table 2). Therefore, this virus did not cause greater nor less axonal involvement if compared to cases without HSV-1.

As to PFP degree, we observed that negative PCR for HSV-1 had a homogenous distribution among grades II to IV. In positive PCR cases, 63% presented grades V or VI. This would indicated that the virus causes greater neural involvement. However, such data is not corroborated by facial nerve electroneurography findings.

Another important fact is patient clinical evolution, assessed according to HSV-1 positiveness. As we observed, positive or negative PCR HSV-1 did not influence patient recovery. This fact was also observed by Furuta et al.^13^

## CONCLUSION

HSV-1 present in the saliva of Bell's PFP patients tells us that viral reactivation may be the very etiology of this disease. Detecting the virus in the saliva does not alter the disease prognosis.

**Chart 1:**
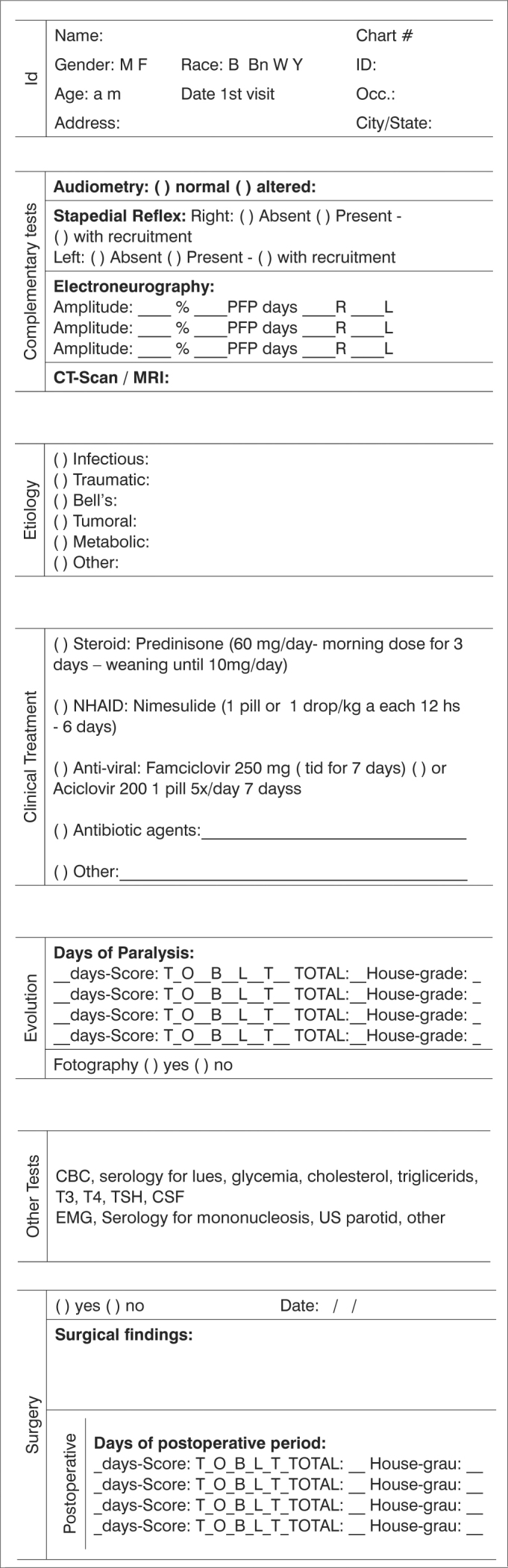
Irmandade da Santa Casa Misericórdia de S.Paulo - Departament of Otolaryngology – Peripheral Facial Paralysis Protocol
